# Atherogenic index of plasma is a novel predictor of non-alcoholic fatty liver disease in obese participants: a cross-sectional study

**DOI:** 10.1186/s12944-018-0932-0

**Published:** 2018-12-13

**Authors:** Qian WANG, Dongmei Zheng, Jia Liu, Li FANG, Qiu Li

**Affiliations:** 1 Department of Endocrinology, Shandong Provincial Hospital affiliated to Shandong University, 324 Jing 5 Road, Jinan, 250021 Shandong Province China; 2 Shandong Clinical Medical Center of Endocrinology and Metabolism, 324 Jing 5 Road, Jinan, 250021 Shandong Province China; 3 Institute of Endocrinology and Metabolism, Shandong Academy of Clinical Medicine, 324 Jing 5 Road, Jinan, 250021 Shandong Province China; 4 Department of Ultrasound, Shandong Provincial Hospital affiliated to Shandong University, 324 Jing 5 Road, Jinan, 250021 Shandong Province China

## Abstract

**Background:**

The atherogenic index of plasma showed to be related with some chronic disease like cardiovascular diseases and atherosclerosis. Body mass index which was commonly used in clinical practice is not an accurate index to predict non-alcoholic fatty liver disease. The aim of this study is to investigate the relationship between atherogenic index of plasma and non-alcoholic fatty liver disease in obese participants.

**Methods:**

538 obese subjects were included in this cross sectional study. Non-alcoholic fatty liver disease was diagnosed by B-ultrasonography after excluding participants with other liver diseases. The atherogenic index of plasma was classified into three groups: the low (< 0.11), the intermediate (0.11–0.21) and the high (> 0.21) risk. The participants were separated into groups according to their atherogenic index of plasma levels. The area under receiver operating characteristic curve of the atherogenic index of plasma for predicting non-alcoholic fatty liver disease was calculated.

**Results:**

There were concordances between increased atherogenic index of plasma and significant increase in the value of body mass index, waist circumference, alanine aminotransferase, glutamyl transpeptidase and lipid profile. The atherogenic index of plasma is strongly associated with non-alcoholic fatty liver disease. Compared to the low risk group, the high risk group had a 5.37 folds risk after adjustment for covariates. Results of receiver operating characteristic curves showed that the area under the curve (95% confidence intervals) was 0.718 (0.670–0.766).

**Conclusion:**

These data suggest that atherogenic index of plasma might be a method which can be used in the auxiliary diagnosis of non-alcoholic fatty liver disease.

## Introduction

Non-alcoholic fatty liver disease (NAFLD), initiated by the accumulation of fat in hepatocytes in the absence of excessive alcohol intake, is currently a common cause of chronic liver disease and has been associated with obesity, type 2 diabetes, hyperlipidemia, and insulin resistance. Throughout the world, the incidence rate of NAFLD is estimated to range from 5 to 30% depending on geographical variations and its incidence rate has rapidly increased in recent decades [1, 2]. NAFLD which was considering as the new global epidemic needs better awareness.

With the prevalent of western diet, obesity becomes a more important medical and social problem. More than half of the people in the US were overweight or even worse. About 30.48% of the people were overweight, and 8.77% were obesity in China [3, 4]. Overweight and obesity are well-established risk factors for diabetes, cardiovascular disease (CVD), certain cancers and premature death, as well as adverse reproductive consequences [5–7].

Although the fact that obesity increases risk of certain diseases is well accepted by clinicians, evidence has shown that some individuals who are obese are also metabolically healthy. Increasing evidence suggests that body mass index (BMI), the most common proxy measure used to help categorize overweight or obesity, is an imprecise measure of body-fat-related risk of non-communicable diseases.

The atherogenic index of plasma (AIP), the logarithm of the molar ratio of triglyceride to high density lipoprotein cholesterol (TG/HDL-C), has shown a strong correlation with size of the low density lipoprotein cholesterol (LDL-C) particle. It has been shown that AIP is a strong marker to predict the risk of atherosclerosis and coronary heart disease [8–10]. Considering of the close relationship between the dyslipidemia and NAFLD, we assume that AIP may be a maker to predict NAFLD, especially in obese people.

The aim of our research is to figure out whether AIP can be a marker to predict the diagnosis of NAFLD in obese people.

## Methods

### Study population

All the eligible participants were over 18 years old and had been living in their current residence for at least 5 years from each community or village. Inclusion criteria were as follows: (1) age ≥ 18 years, (2) BMI ≥28 kg/m2. Exclusion criteria were as follows: (1) missing important information (such as age, sex or ultrasonography), (2) complications or conditions that affected lipid metabolism, (3) taking any drugs that lipid metabolism in the previous three months, and (4) obviously poor compliance. All of them signed informed consent before the examination.

### Data collection

Data was collected at local health stations by trained medical staff. Weight was measured in kilograms while height was measured in centimeters, respectively. Waist circumference (WC) was measured at the umbilicus level with the participants in the standing position. BMI was equal to weight (kg) divided by squared height (m).

Blood samples were collected from all participants after an overnight fast of at least 10 h. The serum lipid parameters, glutamyl transpeptidase (GGT), aspartate aminotransferase (AST), alanine aminotransferase (ALT) and fast plasma glucose (FPG) were measured with the ARCHITECT ci16200 Integrated System (Abbott, Illinois, USA).

### Definitions of NAFLD, AIP stratification and obesity

Diagnosis of fatty liver by ultrasonography is defined by the presence of at least two of three abnormal findings: diffusely increased echogenicity (‘bright’) liver – with stronger echoes than in the renal parenchyma, vascular blurring, and narrowing of the lumen of the hepatic veins [9]. In addition, other liver diseases should be excluded [11]. According to previous studies, AIP was stratified into three groups: low- (< 0.11), intermediate- (0.11–0.21) and high-risk (> 0.21) [9, 12, 13]. Overweight was defined as a BMI of 24.0 to 27.9, and obesity was defined as a BMI of 28.0 or higher [14].

### Data analysis

Gender, age, BMI, WC, total cholesterol (TC), TG, LDL-C, FPG, HDL-C was showed in different groups according to NAFLD or not. Differences between mean values were tested using Student’s t-test. The comparison among groups in Table 1 was determined by one-way analysis of variance (ANOVA).Chi-squared test was used to analyze whether if there were any statistically difference in the prevalence of NAFLD in different AIP quartiles. Logistic regression models were used to analyze the association between AIP and NAFLD. Odds ratios (ORs) were obtained from logistic regression analysis, and the results were presented as ORs with a 95% confidence interval (CI). A *P* value of < 0.05 was defined as statistically significant. The accuracy of using AIP and other related parameters to predict NAFLD was assessed by calculating the non-parametric area under the receiver-operating characteristic (ROC) curve (AUC) with 95% CI. With the limitation of detection means, the criteria of NAFLD used in our research was by ultrasound.

**Table 1:** Clinical characteristics in three groups according to AIP levels

	low	intermediate	high	P
Male (%)	34.0	63.4	69.9	0.000
Age	42.6 ± 11.8	42.2 ± 11.2	41.7 ± 11.5	0.718
BMI	30.48 ± 2.16	30.63 ± 2.03	30.97 ± 2.21	0.058
WC	96.45 ± 8.75	99.22 ± 10.76	100.26 ± 8.68	0.000
AIP	−0.11 ± 0.15	0.16 ± 0.03	0.48 ± 0.28	0.000
TC	5.04 ± 0.93	5.35 ± 1.04	5.45 ± 1.12	0.000
LDL	2.78 ± 0.70	3.13 ± 0.74	3.07 ± 0.74	0.000
HDL	1.25 ± 0.22	1.09 ± 0.19	0.92 ± 0.21	0.000
TG	1.00 ± 0.28	1.57 ± 0.27	3.16 ± 2.20	0.000
ALT	19.82 ± 11.43	38.17 ± 95.01	29.38 ± 39.73	0.000
AST	24.58 ± 6.96	41.87 ± 122.09	28.82 ± 8.72	0.019
GGT	28.94 ± 21.33	40.24 ± 29.03	63.64 ± 71.70	0.000
FPG	5.76 ± 1.33	5.89 ± 1.59	6.07 ± 1.72	0.110

## Results

**Fig. 1:**
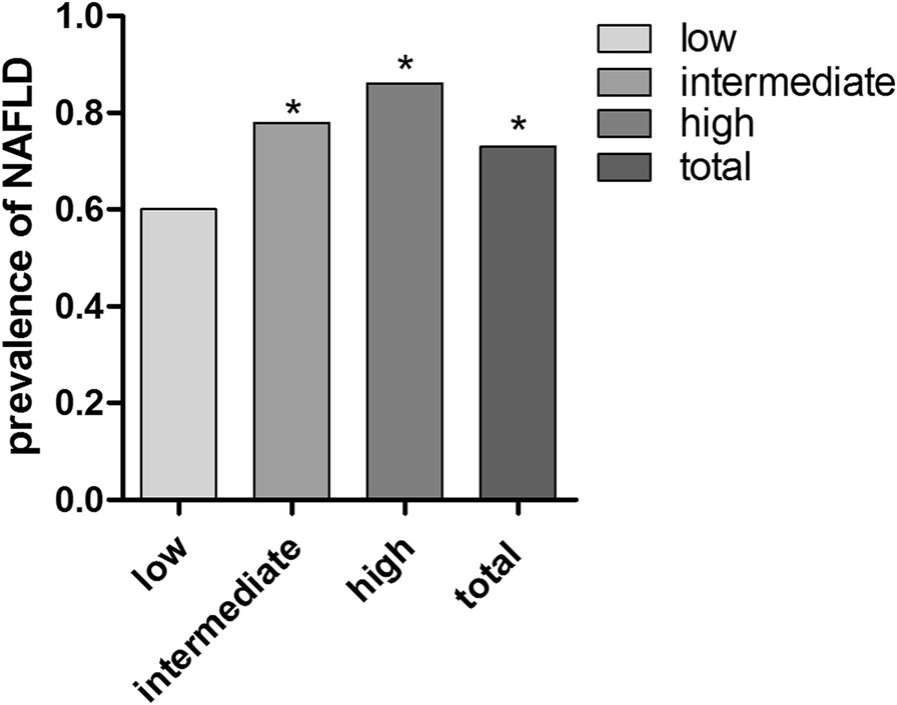
the prevalence of NAFLD according to AIP stratification Lengends: AIP was classified into three levels: low (< 0.11), intermediate (0.11–0.21), high (> 0.21) risk. * means significant difference exists compared to low group

**Fig. 2:**
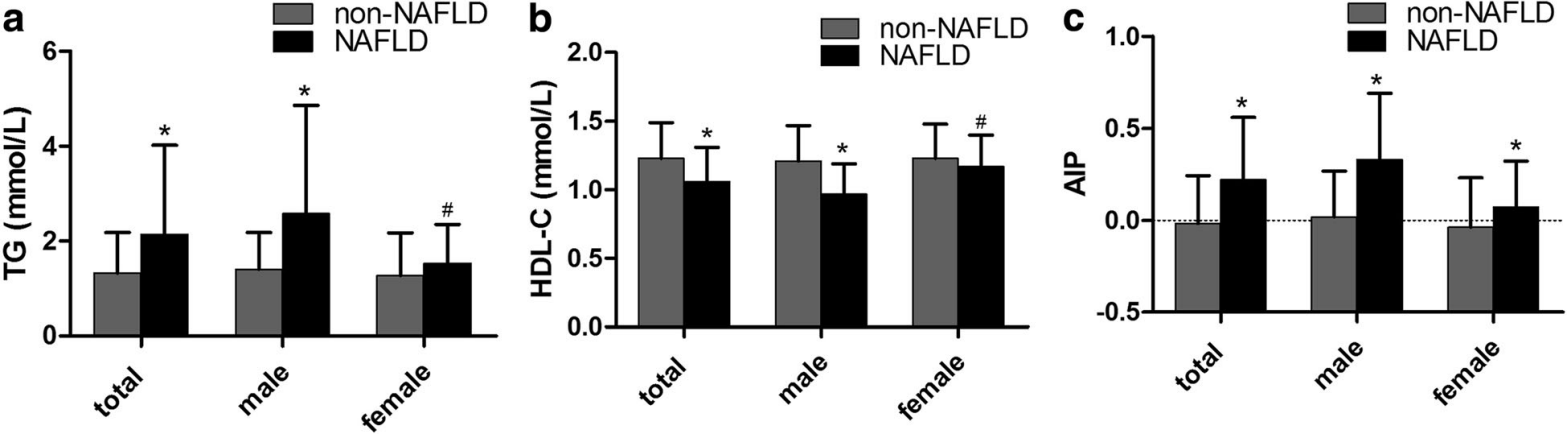
TG, HDL-C and AIP levels according to NAFLD (non-NAFLD participants vs NAFLD participants) in different groups by gender Legends: a. TG (mmol/L); b. HDL-C (mmol/L); c. AIP. Significance levels: * < 0.001, # < 0.05.

A total of 538 obese people were included in the study. Table 1 described the characteristics of the participants. The mean age was 42.18 ± 11.59 years, and 281 (52.23%) were male. The parameters were also described in groups according to AIP stratification: low (*n* = 250, 46.5%), intermediate (*n* = 82, 15.2%), high (*n* = 206, 38.3%). Significant differences were observed in lipid profile, WC, AST, ALT, GGT grouped by AIP. The prevalence of NAFLD was increased in intermediate group and high group. There are significant differences between the low group and other groups in Fig. 1. Figure 2 showed the levels of TG, HDL-C and AIP according to the diagnosis of NAFLD grouped by gender. Significant discrepancy showed in all the three parameters in both genders.

Table 2 shows the association between the anthropometric parameters with NAFLD. When including AIP, TC, LDL-C, ALT, AST, GGT, FPG, gender, age, WC, BMI in the multivariate logistic regression analysis of NAFLD, AIP, TC, LDL-C, GGT, WC, BMI showed a significant relationship with NAFLD. Among these, AIP showed a strong relationship (5.37, 2.26–12.73, *p* < 0.000) with NAFLD.

**Table 2:** Multivariable logistic regression analyses on the association between AIP and NAFLD

	OR	95%CI	P
AIP	5.37	2.26–12.73	0.000
TC	0.47	0.24–0.95	0.036
LDL	3.04	1.18–7.85	0.021
ALT	1.04	1.00–1.08	0.027
AST	0.97	0.94–1.00	0.023
GGT	2.10	1.20–3.67	0.009
FPG	0.99	0.85–1.14	0.842
BMI	1.27	1.11–1.46	0.001
WC	1.05	1.01–1.08	0.005
GENDER	0.59	0.33–1.05	0.070
AGE	0.99	0.97–1.01	0.432

To assess the accuracy of anthropometric parameters to predict the diagnosis of NAFLD, AUC were conducted which were showd in Table 3. Among all the anthropometric parameters, AIP showed to be the best predictor with an AUC of 0.718 in the obese participants we included. Gender difference can be seen from the table. The results in men fitted those of the total participants. However, AUC of AIP was lower than GGT, BMI and WC in female.

## Discussion

There are 3 main finding in the study. First, this research demonstrated that there were concordances between increased AIP and significant increase in the value of BMI, WC, ALT, GGT and lipid profile (Table 1). This finding is similar with the previous study about the association between AIP and cardiovascular diseases. Second, AIP with some other parameters were strongly associated with NAFLD in the multivariate logistic regression. Third, AIP was the best predictor of NAFLD with an AUC of 0.718 compared with other related indexes in the total participants. But in female participants, GGT turned out to be the best (AUC = 0.689) in all the parameters.

A large population-based study found that 91% of obese individuals (BMI > 30 kg/m^2^) had evidence of steatosis on ultrasound [15]. The obese participants in our research were all BMI ≥28 kg/m^2^. The prevalence of NAFLD in our study was 73%, which means that about a quarter of obese people had no fatty liver. Although BMI is one of the most common measurement to categorize overweight or obesity, increasing evidence suggests that BMI is not a precise measure of body fat related risk of non-communicable diseases. Another study also showed AIP to be an important tool in obese participants with cardiovascular disease. More measurements like visceral fat area, inflammatory factors were included in the predictor of NAFLD. But almost all of them were not commonly used in clinical practice. An accurate index is needed to screen and predict NAFLD in clinical applications.

The progression of NAFLD results from an imbalance between lipid uptake and lipid disposal and eventually causes oxidative stress and hepatocyte injury. Although the precise pathological process has not been elucidated, it is been widely accepted that excess TG accumulation in the liver is the prerequisite for NAFLD. TG showed a great correlation with NAFLD in both epidemiologic studies [15] and animal research [16] . HDL-C was significantly decreased in participants with NAFLD in our research (Fig. 2). With the close correlation between TG, HDL-C and NAFLD, we assumed that an index which was calculated with TG and HDL-C may be a predictor for NAFLD. AIP was calculated as log (TG/HDL-C). AIP was used in some research to predict cardiovascular diseases and metabolic syndrome. With being logarithmically transformed, TG/HDL-C could be adjusted for the lack of normative distribution and demonstrate a correlation with smaller LDL-C particles. Compared with the traditional lipid profile, AIP was proved to be a better predictor of atherosclerosis than LDL-C [13, 17].

When we compared some related parameters of NAFLD, AIP showed a better AUC in predicting NAFLD than any other indexes. Previous studies have shown that higher TG/HDL-C indicates insulin resistance, which could be another possible explanation for AIP being a good index considering that insulin resistance is closely related with NAFLD. Another study were in line with our study which were also conducted in obese participants. It was reported that patients with prominent obesity, but with low TG/HDL-C values, had only a low increased risk of coronary heart disease or cardiovascular disease, while patients with high TG/HDL-C had a 70% increased risk, compared to normal weight and low TG/HDL-C as reference. The AUC of WC (0.695) was larger than that of BMI (0.671), which was consistent with another study on the strategies for predicting NAFLD [18]. This result may be explained by the fact that Asians are more likely to have central fat deposition even in a lower BMI. Although GGT seemed to be a good predictor (AUC = 0.703) in our research, few research paid attention on it. It is more related with NAFLD on its severity.

Table 3 showed gender difference in the AUC of AIP for the presence of NAFLD. AIP seemed to be a better predictor in male subjects. The difference was also seen in other studies of NAFLD, which TG and HDL-C showed significant differences for NAFLD in men, while not in women [19, 20]. These results differ from the previous studies from India, in which TG showed a better index in female for NAFLD [21]. These relationships may partly be explained by the effect of different adipose tissue distribution according to gender which played important role in the development of NAFLD. Though subcutaneous adipose tissue is similar in both gender with NAFLD but male store more visceral adipose tissue compared to female, which means BMI could be a rather inaccurate for male subjects. This also fitted with our results that the AUC of BMI is higher than that of AIP in female participants. Another possible explanation for the gender difference may be related with the effect of varying sex hormone on NAFLD [22].

**Table 3:** The area under the receiver operating characteristic curves of AIP, lipid profiles, AST, ALT, GGT, FPG, BMI, WC for the presence of NAFLD classified by gender

	Total			Male			Female		
	AUC OR	95%CI	P	AUC OR	95%CI	P	AUC OR	95%CI	P
AIP	0.718	0.670–0.766	0.000	0.775	0.709–0.841	0.000	0.633	0.561–0.706	0.000
GGT	0.703	0.653–0.753	0.000	0.675	0.598–0.752	0.000	0.689	0.620–0.759	0.000
TC	0.577	0.522–0.631	0.006	0.547	0.459–0.634	0.282	0.588	0.515–0.660	0.020
LDL	0.607	0.552–0.661	0.000	0.570	0.482–0.658	0.106	0.615	0.544–0.687	0.002
HDL	0.309	0.259–0.358	0.000	0.239	0.166–0.313	0.000	0.420	0.347–0.493	0.034
TG	0.698	0.649–0.748	0.000	0.731	0.658–0.803	0.000	0.633	0.561–0.704	0.000
ALT	0.679	0.630–0.728	0.000	0.640	0.559–0.721	0.001	0.635	0.567–0.704	0.000
AST	0.617	0.566–0.668	0.000	0.571	0.490–0.652	0.099	0.575	0.505–0.646	0.046
FPG	0.596	0.544–0.648	0.001	0.600	0.512–0.689	0.045	0.549	0.478–0.619	0.197
BMI	0.671	0.622–0.720	0.000	0.728	0.655–0.800	0.000	0.639	0.571–0.707	0.000
WC	0.695	0.647–0.743	0.000	0.683	0.609–0.757	0.000	0.658	0.590–0.726	0.000

### Limitation

First this was a cross-sectional study and could not definitively provide a causal relationship between AIP and NAFLD. Second, no grades on NAFLD were included in our study. In the future more research should focus on the severity of NAFLD. Third, potential confounders, especially those could affect NAFLD, such as lifestyle and dietary habits, were not included in our study. Last, the diagnosis of NAFLD was not through biopsy but through ultrasonography. However, in the Chinese guideline and most guidelines in other countries, liver biopsy is not recommended but also not applicable with high sensitivity and specificity in all the participants. Ultrasonography is the most applicable and convenient way to diagnosis NAFLD [23].

## Conclusion

In summary, our study showed that AIP were strongly correlated with NAFLD in obese participants. AIP should be used as a regular monitoring index of NAFLD in clinical practice, especially for obese men.

## Abbreviations

AIP: atherogenic index of plasma
ALT: alanine aminotransferase
ANOVA: analysis of variance
AST: aspartate aminotransferase
AUC: non-parametric area under the receiver-operating characteristic curve
BMI: body mass index
CI: confidence interval
FPG: fast plasm glucose
GGT: glutamyl transpeptidase
HDL-C: high density lipoprotein cholesterol
LDL-C: low density lipoprotein cholesterol
NAFLD: non-alcoholic fatty liver disease
OR: odds ratios
ROC: receiver-operating characteristic
TC: total cholestrol
TG: triglyceride
WC: waist circumference
